# Genetic mutations associated with metastatic clear cell renal cell carcinoma

**DOI:** 10.18632/oncotarget.7473

**Published:** 2016-02-18

**Authors:** Zhongjun Li, Ping Hao, Qingjian Wu, Fengjie Li, Jiang Zhao, Kaijin Wu, Cunye Qu, Yibu Chen, Meng Li, Xuelian Chen, Andres Stucky, Jiangjian Zhong, Longkun Li, Jiang F. Zhong

**Affiliations:** ^1^ Department of Blood Transfusion, Second Affiliated Hospital, Third Military Medical University, Chongqing, P. R. China; ^2^ Ostrow School of Dentistry and Department of Pediatrics, School of Medicine, University of Southern California, Los Angeles, CA, USA; ^3^ Department of Oncology, Second Affiliated Hospital, Third Military Medical University, Chongqing, P. R. China; ^4^ Department of Urology, Second Affiliated Hospital, Third Military Medical University, Chongqing, P. R. China; ^5^ Bioinformatics Service, Norris Medical Library, University of Southern California, Los Angeles, CA, USA; ^6^ Z-Genetic Medicine LLC, Temple City, CA, USA

**Keywords:** Plk5, metastasis-specific mutation, renal cell carcinoma

## Abstract

Metastasis is the major cause of death among cancer patients, yet early detection and intervention of metastasis could significantly improve their clinical outcomes. We have sequenced and analyzed RNA (Expression) and DNA (Mutations) from the primary tumor (PT), tumor extension (TE) and lymphatic metastatic (LM) sites of patients with clear cell renal cell carcinoma (CCRCC) before treatment. Here, we report a three-nucleotide deletion near the C-region of Plk5 that is specifically associated with the lymphatic metastasis. This mutation is un-detectable in the PT, becomes detectable in the TE and dominates the LM tissue. So while only a few primary cancer cells carry this mutation, the majority of metastatic cells have this mutation. The increasing frequency of this mutation in metastatic tissue suggests that this Plk5 deletion could be used as an early indicator of CCRCC metastasis, and be identified by low cost PCR assay. A large scale clinical trial could reveal whether a simple PCR assay for this mutation at the time of nephrectomy could identify and stratify high-risk CCRCC patients for treatments.

## INTRODUCTION

Renal cell carcinoma (RCC) accounts for about 2-3% of all cancers and worldwide more than 250,000 new cases each year [1]. The most common type of RCC is clear cell renal cell carcinoma (CCRCC), which originates from the epithelial lining of the proximal convoluted tubules and is responsible for 60% to 80% of RCC among adults [2]. Surgical resection is currently the principal treatment. However, at the time of diagnosis approximately 25-30% patient already present metastasis with a median overall survival of less than 2 years [3]. More than 40% of RCC patients develop metastases after radical nephrectomy with poor survival rate [4]. Despite improved therapeutic regimens, patients with metastatic RCC still have poor prognosis with an average survival of only 6 to 12 months from the time of diagnosis [5]. Therefore, biomarkers associated with metastatic CCRCC are important for early detection and selection of appropriate therapeutic strategies.

Here, we employed a novel strategy with next generation sequencing (NGS) to identify specific mutations as biomarkers for metastatic CCRCC. We compare the genetic profiles from different tumor tissues of the same patient (primary tumor vs tumor extension vs lymphatic metastasis) to identify metastasis-specific mutations. Genomic instability in tumors generates large-scale cellular heterogeneity within a tumor population, from which rare variants evolve through a Darwinian selection process leading to successful metastasis [6, 7]. Consequently, these genetic variants (mutations) will be enriched in the metastatic tissue if they provide growth advantages. Comparing gene expression and mutation profiles among different sites of the same patient, we eliminate individual genetic variations and tumor-specific genetic alternations for better detection of metastasis-specific mutations. With this approach, we identified a small three-nucleotide deletion in the polo box protein binding domain (PBD) of the C-terminal region of Plk5, a promising genetic biomarker for metastatic CCRCC.

Initially identified in *D. Melanogaster,* Polo-Like Kinases (Plk) are a family of evolutionary conserved proteins characterized by their expression of one or more polo box protein binding domain (PBD) near the C terminal and a Ser/Thr kinase near the N-terminal [8]. Plk's are part of a regulatory network, controlling Cdk1/cyclin B complex activation and entry into mitosis at the G_2_/M transition [9]. Among all Plks (Plk 1-5), Plk5 is a DNA damage inducible gene and ectopic expression of Plk5 leads to cell cycle arrest and apoptosis in mice [10]. Human Plk5 is significantly silenced in astrocytoma and glioblastoma multiforme by promoter hypermethylation but expressed in normal brain tissues [11, 12]. The Plk5 mutation reported here could be a variation of Plk5 silencing in metastatic CCRCC. Given the regulatory role of Plk5 in cell cycle, this newly identify metastatic mutation could be an new addition to the known metastasis related genes [13] as an intervention target and biomarker in CCRCC [11, 12].

## RESULTS

### Metastasis related genetic alterations

**Figure 1:**
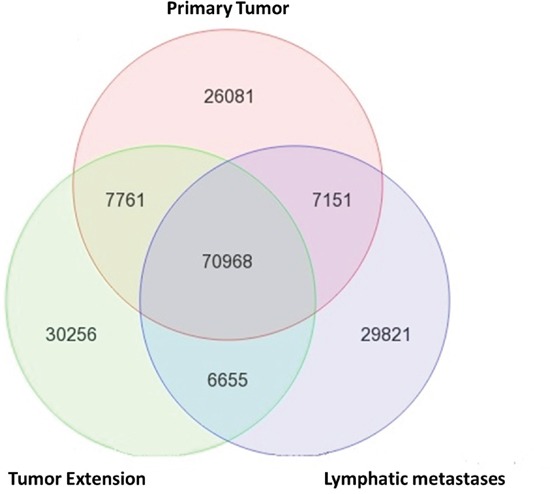
Molecular heterogeneity among tumor cells of the same patient DNA and RNA were extracted from primary tumor (PT), tumor extension (TE), and lymphatic metastases (LM) of a stage-4 CCRCC patient. The genetic alternations detected by both DNA exome-seq and RNA-seq at each tissue were compared. Unique genetic alternations were detected in PT, TE and LM of the same patient and suggest cellular (number of tumor cells) heterogeneity and molecular heterogeneity (mutation profiles) of CCRCC at different locations of a same patient. The 29,821 variants that are unique in the LM were further analyzed for metastatic specific mutations.

Traditionally, metastasis-related genes are identified by comparing differential gene expression profiles between tumor and metastatic cells. Here, we report a novel approach with next generation sequencing (NGS). To identify the mutations that are unique to the metastatic RCC cells, we harvested both DNA and RNA from tissue slides of primary tumor (PT), tumor extension (TE) and lymph node metastasis (LM) from the same patient for NGS on HiSeq 2000 (Illumina, USA). Only tissue slides with >80% tumor cells (examined by a pathologist) were used for DNA and RNA extraction. To avoid sampling variations and improve the reliability of NGS, both DNA and RNA of a sample were extracted from the same tube with TRIzol (Thermofisher, USA). As expected, the majority of genetic alternations (70,968) are common for all three sites because they are from the same patient (Figure 1). However, all sites have unique genetic alternations as well. TE has more unique alternation (30,256) than PT (26,081) and LM (29,821). This data underscore the heterogeneity of tumor samples. Both PT and LM contain a majority of cells already adapted to their local environments. On the other hand, TE has the highest cellular heterogeneity and the cancer cells are under selective pressure in a transitional status from PT to a new environment.

### Differential gene expression among different sites

**Figure 2:**
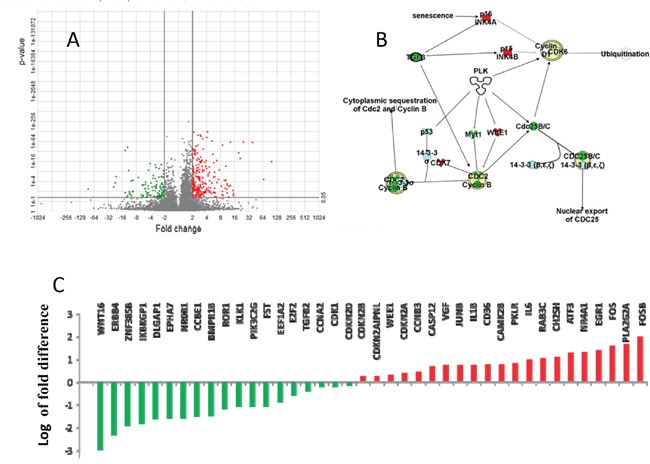
Differential gene expression between primary tumor (PT) and lymphatic metastasis (LM) Pathway analysis of differential expressed genes between PT and LM suggests the involvement of Polo kinases pathway, which is the critical regulator of cell division via chromosomal aggregation and spindle formation. **A.** There are 240 genes differentially expressed between PT and LM, with 111 genes significantly down-regulated in PT (p <0.05). **B.** Majority of differentially expressed genes are involved in the Plk5 pathway. **C.** Many cyclin dependent kinases were significantly up-regulated in LM. FosB, FOS, EGR1, IL6 and VGF are up-regulated 98,36, 23, 9 and 5-folds in LM respectively (Red column). WNT and ERBB4 were down-regulated 16 and 197-folds in LM respectively (Green column). Fold changes are plotted in log scale. Red: up-regulated genes in LM; Green: Down-regulated genes in LM.

With network analysis we examined genes that were differentially expressed between the PT and the LM from the RNA-seq (Figure 2A). We identified genes that are expressed differentially between the PT and LM. Among these differential expressing genes, the most dramatic increase was in early response genes FosB (98 fold, p = 1.55E-16) and Fos (36 fold, p =1.48E-60). Significant up-regulation in metastatic sample was also observed in Early Growth Response genes (EGR). Compared to PT, LM has a 23-fold increase in EGR1 (p =2.12E-62) and a 14 fold increase in EGR3 (p=0.007.38). Ingenuity^®^ Pathway Analysis (IPA^®^) indicated that cyclins and the cyclin dependent kinases (Cdk) were significantly enriched among differentially expressed genes. These differentially expressing genes involve multiple pathways including the Plk cell division pathway (Figure 2B and Figure 2C).

### Plk5 mutation specifically associated with lymph node metastasis

**Figure 3:**
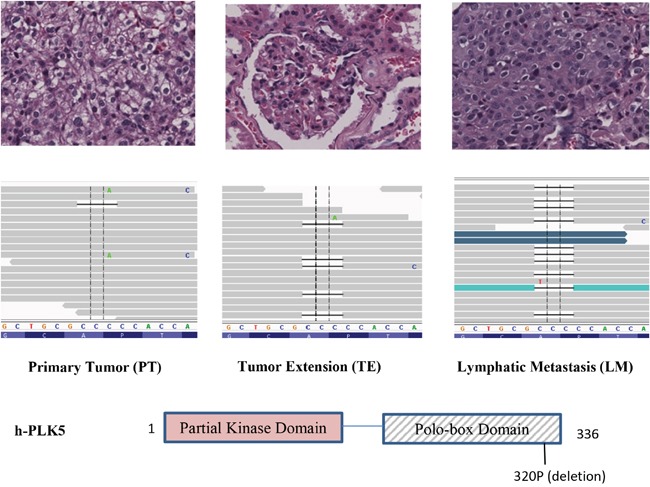
Increasing frequency of a Plk5 mutation in lymphatic metastasis (LM) A deletion in Plk5 was identified with increasing frequency in LM. This deletion is not detectable in primary tumor (PT, left panel), becomes visible in tumor extension (TE, middle panel), and dominate in the lymphatic metastasis (LM, right panel). On top of each panel is the pathology image of CCRCC cells. The increasing frequency of this mutation in LM suggests that cancer cells with this Plk5 mutation have advantages in metastasis tissues and majority of metastatic cells carry this mutation. The mutation is a deletion of a proline of the polo-box domain in Plk5.

We used InterProScan to further search for DNA mutation coordinates that fall within functional protein domains. We identified a small in-frame deletion in exon 14 of Plk5 which dominates in the metastasized RCC samples but not in the primary tumors. Specifically, a three-nucleotide (CCC) deletion at position 1535177 of Chromosome 19 (rs58035688, 320P) was detected with dominant frequency in LM (Figure 3). As indicated by histology slides, samples are considerably heterogeneous with majority of cells are cancer cells (Figure 3). The deletion is not detectable in PT (only one read). In the TE, this deletion is detected because some of the cells carry this mutation. In the LM, the deletion frequency is the highest with the majority of reads containing the deletion. This result suggested that majority of metastatic CCRCCs carry this mutation which is rare in the primary tumor.

### Differential expression of Plk5 protein

**Figure 4:**
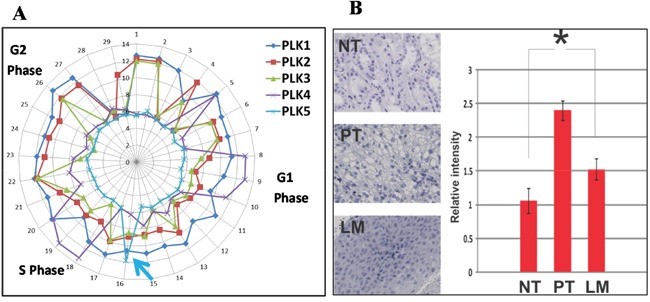
Plk5 mRNA perturbation during cell cycle and its protein expression support that Plk5 is a tumor suppressor **A.** With microfluidic devices, transcriptomes of individual Hela cells were arranged by similarity to reveal the perturbations of individual genes at different stages of a cell cycle. The mRNA perturbation of Plks (Plk1-5) during a cell cycle is plotted along a cell cycle. Unlike Plk1-4, Plk5 mRNA is highly expressed right before entering S phase and rapidly down-regulated (blue arrow). This perturbation pattern is typical for S-phase check point genes. **B.** Agreeing with previous studies, immunostaining shows that Plk5 is induced by DNA damage in the primary tumor (PT) when compared with normal tissue (NT). However, Plk5 protein is less at lymphatic metastasis (LM) suggest more cells lost the Plk5 check-point before entering S phase. Relative intensity is quantified with Imagej.

This in-frame Plk5 deletion (rs58035688) was reported with unknown function in the “1000 Genomes” project. Previously, the anti-proliferative effect of murine and human Plk5 protein has been reported [11] and DNA damage induce the expression of Plk5 protein [10]. These studies suggest that Plk5 is a tumor suppressor. Therefore, we first examine the mRNA perturbation profiles of all Plks in a Hela cell line. With microfluidic technology, we have previously obtained single-cell transcriptomes from individual Hela cells which are in different cell cycle stages [14, 15]. Because transcriptomes from consecutive cell cycle stages are more similar than these from disparate stages, arranging these single-cell transcriptomes by similarity reveals the sequential perturbation of all genes during cell cycle [15, 16]. The mRNA expression of Plks (Plk1-5) in Hela cells during cell cycle were plotted (Figure 4A). The mRNA perturbation profile of Plk5 is different from those of Plk1-4. In agreement with previous studies, [10, 11] the mRNA of Plk5 is rapidly up-regulated before entering S phase, and then rapidly down-regulated. The mRNA perturbation profile of Plk5 suggests that Plk5 is a S phase check-point gene.

The expression of Plk5 protein in HEK295, a human embryonic kidney cell line has been reported previously [10]. Therefore, we performed immunostaining to measure Plk5 protein expression among the different collected CCRCC tissues. In accordance with previous studies [10, 11], Plk5 is expressed higher in cancer cells of PT (Figure 4B). The expression level of Plk5 protein is lower in metastatic CCRCC and slightly higher than that in normal kidney tissues. This result of decreasing Plk5 protein expression in the lymphatic metastatic sites (LM) suggests that the mutation reduced the Plk5 protein levels in LM.

### Plk5 mutation association with metastatic CCRCC in multiple patients

**Figure 5:**
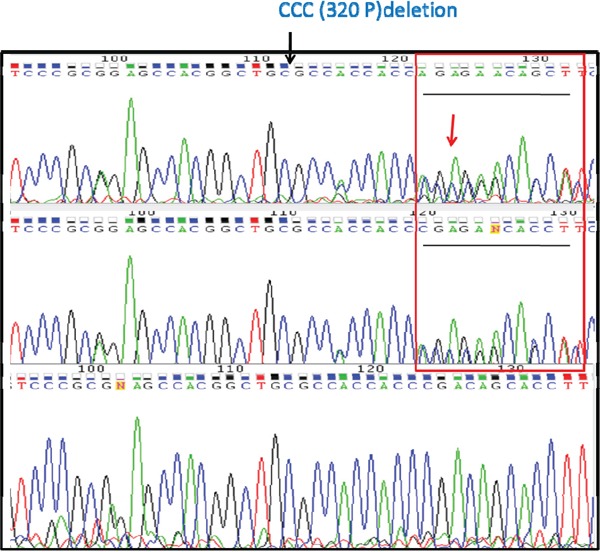
Target sequencing with DNA from CCRCC patients confirms that the Plk5 mutation is metastasis-specific Plk5 was amplified with PCR from genomic DNA of 5 stage-4 CCRCC patients and subjected to Sanger sequencing for confirmation of the rs58035688 deletion. Representative chromatograms from sequenced primary tumor site (PT, Top), the tumor extension (TE, Middle), and the lymphatic metastasis site (LM, Bottom) reveal metastatic specific pattern of the deletion. The deletion is heterozygosis in PT and TE as indicated by the sequence overlap at position 125 in the graph (top and middle). In contrast, the deletion is homozygous in LM. This data suggested that only some CCRCC cells in PT and TE carry this deletion, but majority of CCRCC cells in LM carried this deletion.

To rule out the patient specific possibility, we further performed target sequencing of Plk5 with DNA from other stage-4 CCRCC patients. The sequencing data confirmed that the Plk5 mutation (rs58035688) is predominating in metastatic CCRCC, but not detectable or barely detectable in the primary tumor (Figure 5). This result suggests that this mutation of Plk5 could be used as a biomarker for detecting metastatic CCRCC. A larger scale study with more patient samples is currently underway to confirm early detection of metastatic CCRCC by measuring Plk5 deletion frequency in a sample.

## DISCUSSION

Lymphatic metastasis is the most common way of dissemination of cancer cells. Biomarkers associated with lymphatic metastasis could be a valuable and powerful tool for detection and intervention of metastasis, the major cause of cancer death. Several metastasis related genes (e.t VHL, G250, SETD2, BAP1, PBRM1, p53) in cancer have been reported [13], and targeted therapies have shown promising value on improving cancer treatment. Although acquired mutations have been documented in several types of kidney cancer, mutations associated with metastatic kidney disease remain elusive. The major hurdle for identifying metastasis-associated genes is the heterogeneity of tumor samples which often contain normal cells. Here, we circumvent this hurdle with NGS to identify increasing mutation frequency of a Plk5 mutation (rs58035688) which is enriched in LM of a CCRCC patient. Consistent with previous study [10, 11], cell cycle perturbation profile of Plk5 mRNA suggests that Plk5 is a tumor suppressor at the S phase check-point. Subsequent immunoassays and target sequencing show that this Plk5 mutation is also uniquely associated with lymphatic metastatic CCRCC in other patients.

Accumulating literature associates Plk function abnormalities with malignancy in various types of cancer [12]. Unlike other Plks (Plk1,2,3 and 4), the function of Plk5 in cancer development is not yet well understood, but it was recently shown to localize in the nucleus in response to DNA damage [10] and was silenced in brain tumor tissues [11, 12]. Our study agrees with these studies, the mRNA perturbation profiles during cell cycle suggest that Plk5 prevents cells entering S phase. The higher expression of Plk5 in primary tumor could be a response to the DNA damages, but Plk5 silencing by promoter hypermethylation [10–12] or mutation in the PBD domain to disrupt the expression (as we reported here) allows tumor cells to entering S phase for proliferation disregard their DNA damages. This Plk5 deletion may allow cancer cells to escape from cell cycle arrest in a metastatic site that can potentially induce oxidative stress and DNA damage as shown in a recently reported study [17]. The Plk5 deletion (rs58035688) reported here could be used as a biomarker for early detection of CCRCC lymphatic metastasis, and be an intervention target for preventing metastasis in CCRCC. Albeit this mutation had been previously identified (1000 genome project: rs58035688) this is the first study that directly implicates it to RCC metastasis, and provides evidence that it plays a role in S phase check-point. Screening of this short deletion in Plk5 is a potentially rapid and low cost method that could significantly improve current diagnostic method for high-risk CCRCC patients.

## MATERIALS AND METHODS

### DNA and RNA preparation

Fresh tissues from primary tumors, tumor extension, and metastatic lymph nodes were obtained at surgery from all cases with IRB approval and patient consent. Surgical specimen isolation and handling was done in compliance to standards of clinical care. Each specimen was cut into two parts. One part was used for routine pathological diagnosis, and the other half was frozen in liquid nitrogen for molecular analysis (DNA and RNA extraction). The diagnosis of CCRCC and stage of diseases was confirmed by independent pathologists. After identifying tumor cells by a pathologist, genomic DNA and mRNA were isolated from slides with 80% or more tumor cells by using TRIzol^®^ reagent (Life technologies, USA).

### Library construction and sequencing

For each sample, 1μg of gDNA and 5μg of RNA was prepared and submitted for sequencing. The DNA and RNA quality was evaluated and libraries were constructed with a library construction kit (Illumina, USA). Libraries were sequenced on the Illumina HiSeq 2000 platform (Illumina, USA). The raw reads generated were filtered according to sequencing quality and with regard to adaptor contamination and duplicated reads. Thus, only high-quality reads were remained and used in the genome assembly. Both RNA-seq and Exome-seq data were analyzed with Partek Flow version 4 (Partek Inc., USA). Bases with Phred score less than 20 were trimmed from both ends of the raw sequencing reads, and trimmed reads shorter than 25 nt were excluded from downstream analyses. Both pre- and post-alignment QA/QC was carried out with default settings as part of Flow workflow.

### Gene expression analysis

For RNA-seq samples, trimmed reads were mapped onto human genome hg38 using Tophat 2.0.8 as implemented in Flow with default settings, and using Gencode 20 annotation as guidance. Gencode 20 annotation (www.gencodegenes.org) was used to quantify aligned reads to genes/transcripts using Partek E/M method. [18] Read counts per gene in all samples were normalized using Upper Quartile normalization [19] and analyzed for differential expression using Partek's Gene Specific Analysis method (genes with less than 10 reads in any sample were excluded).To generated a significantly differentially expressed genes among different tissues of the same patient, a cutoff of FDR adjust p<0.05 (Poisson regression) and folder change >|2| was applied.

### Mutations profiling

For Exome-seq samples, trimmed reads were mapped onto human genome hg38 using BWA-MEM 0.7.9a [20] as implemented in Flow with default settings. The aligned reads of each sample were then used to call variants among the samples using Samtools [21] with default settings as implement in Flow. Identified variants were visually inspected using the Integrative Genomics Viewer [22]. We utilized BioBase to identified putative oncogenes and variants that have been singled out as cancer risk genes, which compares the variation sites against publicly available HGMD^®^ (professional, 2015), COSMIC v71, GWAS (February 17 2015) EVS for known exon variants (cESP6500) ClinVar (2015-02), the pharmaco Genomic Mutation Database 2015.1(beta), Allele frequency from 1000 Genomes (dbSNP141) and dbNSFP for non-synonymous functional prediction (v2.9). Before constructing the analysis set, we removed dubious short reading frames and obviously unrelated genes resulting from the filtering parameters.

### Immunohistochemistry

Frozen tumor samples from patients were fixed in 4% PFA for 24 hours, followed by paraffin block embedding and sectioning. Formalin fixed sections are cut at 4 uM and baked at 60 degree for at least 1 hour before deparaffinization in xylene. Then they were washed with 100% ethanol, followed by rehydration in 95% ethanol for 5-10 minute each. The slides are put in 3% hydrogen peroxide in absolute methanol in order to quench endogenous peroxidase. After washing the slides in distilled water, antigen retrieval is performed using citrate buffer (pH 6) and microwaving for 30 minutes. After cooling off at room temperature for 20 minutes, the slides are then blocked with normal serum for 20 minutes to block the non-specific binding. Primary antibody against the Plk5 N terminal region (Abcam, USA) were added and left on the slides for 30 minutes. The slides are washed with PBS for 10 minutes. Chromogen of 0.03% diaminobenzidine is applied for 10 minutes and Hematoxylin is used as the counter stain. The slides are de-hydrated and cover slipped before imaging analysis. Relative intensity of Plk5 protein expression is quantified by Imagej [23].

### Targeted mutation sequencing

Primers for exon 14 of Plk5 were designed using NCBI primer designed tools. A 307 nucleotide sequence was PCR amplified with a forward primer (CTTTGCAGGTGAGCTTCAGT), and a reverse primer(GGTTGAACAGTCATGCCACA). The resulting PCR products were confirmed with a 1.4% agarose gel for fragment size. DNA was then column purified (zymo^®^, USA) and the quality and concentration of the resulting DNA was tested on a nanodrop before sequencing by GENEWIZ.
